# Provider implicit and explicit bias in person-centered maternity care: a cross-sectional study with maternity providers in Northern Ghana

**DOI:** 10.1186/s12913-023-09261-6

**Published:** 2023-03-14

**Authors:** Patience A. Afulani, Jaffer Okiring, Raymond A. Aborigo, Jerry John Nutor, Irene Kuwolamo, John Baptist K. Dorzie, Sierra Semko, Jason A. Okonofua, Wendy Berry Mendes

**Affiliations:** 1 Department of Epidemiology and Biostatistics, University of California, San Francisco, 550 16th St, San Francisco, CA 94158 USA; 2 Infectious Diseases Research Collaboration, Kampala, Uganda; 3 Navrongo Health Research Centre, Navrongo, Ghana; 4 Department of Family Health Care Nursing, University of California, San Francisco, San Francisco, USA; 5 Department of Psychology, University of California, Berkeley, Berkeley, USA; 6 Department of Psychiatry and Behavioral Sciences, University of California, San Francisco, San Francisco, USA

**Keywords:** Person-centered care, Maternity care, Implicit bias, Explicit bias; equity

## Abstract

**Background:**

Person-centered maternity care (PCMC) has become a priority in the global health discourse on quality of care due to the high prevalence of disrespectful and lack of responsive care during facility-based childbirth. Although PCMC is generally sub-optimal, there are significant disparities. On average, women of low socioeconomic status (SES) tend to receive poorer PCMC than women of higher SES. Yet few studies have explored factors underlying these inequities. In this study, we examined provider implicit and explicit biases that could lead to inequitable PCMC based on SES.

**Methods:**

Data are from a cross-sectional survey with 150 providers recruited from 19 health facilities in the Upper East region of Ghana from October 2020 to January 2021. Explicit SES bias was assessed using situationally-specific vignettes (low SES and high SES characteristics) on providers’ perceptions of women’s expectations, attitudes, and behaviors. Implicit SES bias was assessed using an Implicit Association Test (IAT) that measures associations between women’s SES characteristics and providers’ perceptions of women as ‘difficult’ or ‘good’. Analysis included descriptive statistics, mixed-model ANOVA, and bivariate and multivariate linear regression.

**Results:**

The average explicit bias score was 18.1 out of 28 (SD = 3.60) for the low SES woman vignette and 16.9 out of 28 (SD = 3.15) for the high SES woman vignette (*p* < 0.001), suggesting stronger negative explicit bias towards the lower SES woman. These biases manifested in higher agreement to statements such as the low SES woman in the vignette is not likely to expect providers to introduce themselves and is not likely to understand explanations. The average IAT score was 0.71 (SD = 0.43), indicating a significant bias in associating positive characteristics with high SES women and negative characteristics with low SES women. Providers with higher education had significantly lower explicit bias scores on the low SES vignette than those with less education. Providers in private facilities had higher IAT scores than those in government hospitals.

**Conclusions:**

The findings provide evidence of both implicit and explicit SES bias among maternity providers. These biases need to be addressed in interventions to achieve equity in PCMC and to improve PCMC for all women.

**Supplementary Information:**

The online version contains supplementary material available at 10.1186/s12913-023-09261-6.

## Background

Person-centered maternity care (PCMC) refers to care during childbirth that is respectful and responsive to women’s preferences, needs, and values [1, 2]. PCMC emphasizes the continuum of people’s experience of care during childbirth including responsive and supportive care, dignified and respectful care, effective communication, and respect for people’s autonomy. It is a measure of respect for people’s human rights as well as a measure of the quality of care [1, 3–5]. PCMC is a more inclusive term that captures other terminologies used in maternal health such as respectful maternity care and compassionate care. Other terms such as mistreatment, disrespect and abuse, and dehumanized care represent poor PCMC.

Several studies globally have documented a high prevalence of disrespectful, abusive, and neglectful treatment of women during facility-based childbirth [6–9]. Such poor PCMC leads to lack of, delayed, inadequate, unnecessary, or harmful care [10]. Mistreatment deters women from giving birth in health facilities; the experience of poor PCMC, even by a few women, leads to negative community perceptions of quality of care, which discourages other women from giving birth in health facilities [11–14]. On the other hand, positive healthcare experiences can improve health outcomes through pathways such as patient engagement, safety, trust, higher patient and provider satisfaction, and improved psychosocial health,[15, 16]. Further, core components of PCMC such as birth companionship is associated with improved birth outcomes such as shorter duration of labor, decreased caesarean and instrumental vaginal birth, and higher five-minute Apgar scores, which decreases need for neonatal resuscitation [17, 18]. Recent studies have also linked PCMC to improved postpartum outcomes such as lower risk of reporting maternal complications, screening positive for post-partum depression, and reporting newborn complications [19, 20]. Poor PCMC therefore undermines health gains for mothers and babies [10].

Studies in sub-Saharan Africa (SSA) have shown significant gaps in PCMC as evidenced by disrespect and abuse, poor communication, lack of respect for women’s autonomy, and lack of supportive care during prenatal care and childbirth [8, 12, 21, 22]. One study in Ghana, Guinea, Myanmar, and Nigeria found that 42% (*N* = 2016) of women observed were physically or verbally abused, and 35% (*N* = 2672) of women interviewed reported mistreatment such as stigma and discrimination during childbirth in healthcare facilities [9]. Another study using a continuous measure of PCMC with scores ranging from 0–100 (higher scores indicative of higher PCMC) found average PCMC scores below 70 among women surveyed in Ghana, Kenya, and India [8]. The lowest scores were in the communication and autonomy domains, with 60% of women in the Ghana sample reporting providers never explained the purpose of examinations or procedures and 44% reporting providers never asked for their consent before exams and procedures. Over 80% of women in the Ghana sample also reported providers never introduced themselves before attending to them. Prior research also shows disparities in PCMC, especially by socioeconomic status (SES). Across several quantitative studies, women of low SES (measured variously by wealth quintiles, education, literacy, age, and empowerment measures) tend to report poorer PCMC than women of higher SES [8, 9, 21, 23–25]. These disparities are also highlighted in qualitative studies where women reported being discriminated against based on their status, which influences their decision-making on where to give birth [7, 12, 26–28]. We hypothesized that provider biases may be reinforcing these patterns of abuse against low SES women [29, 30].

Bias can be explicit/conscious or implicit/unconscious [31]. Explicit bias refers to conscious attitudes, beliefs, and perceptions about a group, often manifesting as discrimination—the act of treating people differently according to their perceived group [32]. Implicit bias on the other hand operates at an unintentional level and does not require a person to endorse or devote attention to its expression. Instead, it can be activated quickly and unknowingly by situational cues such as a person’s skin color, accent, clothes, or other outwardly appearance [31, 33]. Implicit bias is prevalent in every society, although the content (e.g., stereotypic associations) of biases may differ across contexts [34, 35]. While people may have similar explicit and implicit stereotypes, there is often little correlation between measures of explicit and implicit bias as reporting on explicit bias is prone to social desirability bias [36–39]. Most studies on implicit and explicit bias in health care settings have been conducted in the United States (US), with racial bias commonly studied. For example, several studies have documented anti-Black bias among physicians contributing to lower likelihood of evidence-based prescribing and lower quality interpersonal care for Black compared to White patients [40–42]. SES bias is, however, likely a key bias in context where the predominant bias is not racial.

Within healthcare in the US, SES bias is prevalent and influential [43]. Research with physicians supports this claim, finding that low-SES patients are perceived to be less intelligent, less compliant, and less interested in promoting their own health relative to higher-SES patients [44, 45]. Such differential attitudes by patient SES translate into physician behavior with low-income patients receiving shorter consultations and fewer medical tests than patients with higher income [46]. From the patient perspective, SES bias is felt. Patients report that quality of treatment provided, access to care, and patient-provider interactions is affected by their status, offering evidence of the role of implicit bias [47]. Patients also report experiencing discrimination due to their status, suggesting the persistence of explicit or implicit bias in healthcare [48]. SES bias experienced in interactions with healthcare professionals contributes to distrust and lack of treatment adherence [49], thereby contributing to poorer health over time [50]. Though provider bias based on patient’s SES has been less thoroughly studied in other settings [51], disparities in person-centered care in the healthcare system based on patient SES have been observed worldwide. For example, a study of several countries in Europe found that compared to low SES patients (measured by patient education), high SES patients in Spain, Italy, and France experienced shorter waiting time for specialist consultations [52]. In low and middle-income settings, several qualitative studies have documented preferential treatment of higher SES patients compared to low SES patients, suggesting a role of provider bias [12, 53–56]

Studies on bias in PCMC in Africa are limited. To our knowledge only one prior study empirically examined the role of both provider implicit and explicit bias on PCMC in SSA. This work in Kenya provided initial empirical evidence for the role of both implicit and explicit bias in PCMC disparities by socioeconomic status in SSA [21, 39, 55, 57]. This manifested as providers’ reactions to women's appearances, assumptions about who is more likely to understand or be cooperative, and perceptions of women's expectations and attitudes. These factors, including women's ability to advocate for themselves or hold providers accountable interact to produce PCMC disparities [39]. There is also limited research on factors that may be associated with provider bias. Prior research however suggests that while implicit bias may be similar among providers in the same contexts, some provider factors such as education may be associated with explicit bias [36, 39]. In this study, we sought to extend the evidence base for the role of both explicit and implicit bias in PCMC disparities by socioeconomic status using data from another setting in SSA. This study included a larger sample of maternity providers in Ghana, which provides more statistical power to assess various associations. The primary aim of the study was to assess the extent of provider implicit and explicit SES bias that may contribute to disparities in PCMC in Ghana. A secondary aim was to identify provider and facility-level factors associated with these implicit and explicit SES biases.

## Methods

### Design, participants, setting, and data collection

The data are from a cross-sectional study with healthcare providers who work in maternity units in the Upper East region (UER) of Ghana. The setting and data collection procedures have been previously described [58] and are briefly summarized here. The UER is one of poorest regions in Ghana. The literacy rate (proportion of people from age 6 who can read and write) for the region is about 48% compared to the national average of about 70%. About 37% of the population aged 3 and older have never been to school, and of those over 15 years who have some schooling, less than 20% have more than a secondary school education [59]. The region is divided into 15 administrative municipalities/districts, of which 10 have district hospitals. The doctor-patient ratio for the region is about 1:27,652 and the nurse-patient ratio is about 1:500 [60].

We recruited a total of 150 Providers from the 19 highest volume delivery health facilities (most with an average of 75 births per month or more in the prior year) across the 15 districts in the region from October 2020 to January 2021. There are about 94 high volume delivery facilities across the region (hospitals and health centers that conduct at least 100 deliveries per year). All providers who worked in maternity units in the selected facilities for a minimum of six months at the time of the survey, inclusive of doctors, medical assistants, midwives, nurses, and support staff, were eligible to participate. Two trained research officers (one male and one female) conducted the interviews at private locations at the health facilities or elsewhere based on the provider preference. With approval from the Regional Director of Health Services and permission from leadership of the various health facilities, providers designated to the maternity unit who were available at the time of the visit were invited to participate in the study. The response rate was 80%. Ethics approval was obtained from the Navrongo Health Research Center and the University of California, San Francisco Institutional Review Boards, with additional approval from the UER Director of Health Services. All participants provided written informed consent following receipt of information about the study. All the interviews were conducted in English using a structured questionnaire in the REDCap mobile application [61], and lasted about one hour. The questionnaire included several questions related to explicit bias and provider and facility characteristics. Following the interview, each respondent took a computer-based implicit bias test described below.

### Measures

The measures of implicit and explicit bias used have been previously described [39] and briefly summarized below:

*Explicit bias* was assessed using providers’ perceptions of women’s PCMC expectations and behaviors based on SES, preference for low and high SES women, and a feeling of connection to low and high SES women. Two vignettes (Table 1) were read in counter-balanced order to each provider, followed by ten questions. The first eight questions assessed providers’ perceptions of the woman in the vignette’s expectations for introductions, consenting, and companionship; potential to cooperate, understand explanations, exaggerate pain, and to litigate; as well as provider behavior needed to convey seriousness and gain cooperation. Response options ranged from strongly disagree to strongly agree on a 4-point scale (Table 2). Two final questions asked providers to what extent they would want to be a provider for the woman in the vignette and how connected they felt to her on a scale of 1 to 10. All participants responded to these questions about both the low-SES and high-SES woman. The development of this measure was informed by measurement of explicit bias in prior literature [37, 40] and prior research in Kenya [55]. It was piloted with five providers in Ghana prior to the study.

**Table 1:** Vignettes to assess explicit bias

***Scenario 1: Woman with markers of low SES****:* A 30-year-old poor farmer from one of the villages in the county is admitted to the ward. She dropped out of school in primary two and cannot read or write. She is not covered by insurance and attended ANC only once. She looks very unkempt and did not bring anything with her to be used for the delivery. She presented in labor with her mother-in-law and is complaining of severe abdominal pain. Thinking about this patient: How strongly do you agree/disagree with these statements?
***Scenario 2: Woman with markers of high SES****:* A 30-year-old woman who is the wife of a doctor in the hospital is admitted to your ward. She also works at the local bank and is covered by private health insurance. She received ANC 6 times during her pregnancy. She is very well dressed and has come with all the required items for her labor. She presented in labor with her mother-in-law and is complaining of severe abdominal pain. Thinking about this patient: How strongly do you agree/disagree with these statements?
Statements
1. She is not likely to expect providers to introduce themselves to her 2. She is not likely to understand any explanations 3. Since she has come to the facility, it means she has consented to all examinations and treatments 4. She is likely exaggerating her pain 5. She will not need a companion to stay with her 6. The provider needs to be stern for her to understand the seriousness of the situation 7. She is likely going to be uncooperative when it is time to push and need to be physically restrained 8. She is likely to sue you if something goes wrong 9. On a scale of 1 to 10, where 1 represents lack of connection or warm feelings towards the patient and 10 represents strong connection or strong feelings of warmth towards the patient how connected or warm are you likely to feel towards this patient? 10. To what extent do think you will want to be a provider for patients like her?

**Table 2:** Distribution of responses to the individual questions in the vignettes

Statement	Response	Vignette: N (%)		
		Low SES (*n* = 148)	High SES (*n* = 148)	*p*-value
Not likely to expect providers to introduce themselves to her	Strongly disagree	21 (14.2)	25 (16.9)	< 0.001
	Disagree	58 (39.2)	92 (62.2)	
	Agree	57 (38.5)	21 (14.2)	
	Strongly agree	12 (8.1)	10 (6.7)	
Not likely to understand any explanations	Strongly disagree	31 (21.0)	48 (32.4)	< 0.001
	Disagree	59 (39.9)	70 (47.3)	
	Agree	35 (23.7)	25 (16.9)	
	Strongly agree	23 (15.5)	5 (3.4)	
Has come to the facility, it means she has consented to all examinations	Strongly disagree	21 (14.2)	17 (11.5)	0.772
	Disagree	57 (38.5)	56 (37.8)	
	Agree	44 (29.7)	44 (29.7)	
	Strongly agree	26 (17.6)	31 (21.0)	
Likely to exaggerate her pain	Strongly disagree	31 (21.0)	24 (16.2)	0.004
	Disagree	83 (56.1)	62 (41.9)	
	Agree	23 (15.5)	49 (33.1)	
	Strongly agree	11 (7.4)	13 (8.8)	
Will not need a companion to stay with her	Strongly disagree	43 (29.1)	46 (31.1)	0.251
	Disagree	94 (63.5)	96 (64.9)	
	Agree	7 (4.7)	6 (4.0)	
	Strongly agree	4 (2.7)	0 (0.0)	
Provider needs to be stern for her to understand the seriousness of the situation	Strongly disagree	24 (16.2)	21 (14.2)	0.818
	Disagree	57 (38.5)	60 (40.5)	
	Agree	51 (34.5)	47 (31.8)	
	Strongly agree	16 (10.8)	20 (13.5)	
Likely going to be uncooperative when it is time to push and need to be restrained	Strongly disagree	27 (18.2)	25 (16.9)	0.277
	Disagree	84 (56.8)	72 (48.6)	
	Agree	22 (14.9)	34 (23.0)	
	Strongly agree	15 (10.1)	17 (11.5)	
Likely to sue you if something goes wrong	Strongly disagree	14 (9.5)	1 (0.7)	< 0.001
	Disagree	50 (33.8)	4 (2.7)	
	Agree	58 (39.2)	55 (37.2)	
	Strongly agree	26 (17.6)	88 (59.5)	
Would like to be a provider for this patient	Not at all	3 (2.0)	2 (1.4)	0.046
	A little	27 (18.2)	47 (31.8)	
	Very much	118 (79.7)	98 (66.2)	
			1 (0.7	
Feeling connected to patient on a scale of 0 to 10. *N* = 149	Mean (SD)	7.7 (1.9)	7.5 (1.7)	0.246

*Implicit bias* was measured using an *Implicit Association Test (IAT)* implemented in Inquisit Lab version 5 [62], which was first developed in a study in Kenya [39]. The IAT is a cognitive-behavioral test that measures the strength of automatic associations between concepts in people’s minds based on a sorting task [63]. It has been shown to be a valid and reliable way of measuring implicit bias based on various factors such as race, gender, SES, religion, etc., [36, 64]. The IAT used for this study assessed associations between women’s SES characteristics and providers’ perceptions of women as ‘difficult’ or ‘good’ [55, 65]. Attributes of ‘good’ patients used were likable, cooperative, respectful, intelligent, and responsible; whereas attributes of ‘difficult’ patients were irresponsible, uncooperative, rude, annoying, and stupid. High SES descriptors were wealthy, well-educated, well-dressed, and a banker; low SES descriptors included poor, uneducated, old/torn clothes, and a cleaner. An individual’s IAT score represents the difference in the average length of time they took to sort words during various sections of the test. It is assumed that people will more quickly sort words they associate together than those they do not. IAT scores vary between -2 and + 2. In this study, a positive score indicates a stronger association between high status and good patient and between low status and difficult patient. Increasing positive scores can thus be interpreted as stronger implicit bias in favor of high SES patients. A negative score indicates a stronger association between high status and difficult patient and low status and good patient—implying implicit bias in favor of low SES patients. The IAT has been used in prior studies to assess implicit bias in healthcare settings [40, 42].

### Statistical analysis

Initial analysis included descriptive statistics to characterize the sample and measures and factor analysis to assess the psychometric properties of the composite measures. We then generated explicit bias scores by summing responses to the questions for each vignette. We used mixed-model ANOVA to assess if responses based on the two SES vignettes differed. For implicit bias, we used dependent samples t-test to test whether the average IAT score differed significantly from zero—zero indicating no bias. We then analyzed the associations between bias measures and provider and facility characteristics using cross-tabulations and bivariate linear regressions with robust standard errors; and multivariate associations using multilevel linear regressions. In model building, we included all variables with *p*-values of 0.2 and below from the bivariate analysis to minimize negative confounding, and those with known relationship with the outcome of interest. We then systematically removed non-significant variables from the model until the best fit was attained using the Akaike information criteria. We used STATA version 14.1 for all analysis (College Station, TX).

## Results

### Demographics

Of the 150 providers who participated in the interviews, most were female (97.3%), married (74.0%), between 30 and 52 years of age (71.3%), and were nurses or midwives (97%). No doctor participated. About two thirds worked in government hospitals (61.4%), with 21.3% working in government health centers, and 17.3% in Mission/private facilities. Close to half (46.0%) had been in their positions for five years or less (Table 3).

**Table 3:** Participant characteristics

Characteristic	Category	Survey (*N* = 150)
		No. (%)
Facility type	Govt hospital	92 (61.4)
	Govt health center/Dispensary	32 (21.3)
	Mission/private	26 (17.3)
Position	Nurse/Midwife	145 (96.7)
	Support	5 (3.3)
Gender	Male	4 (2.7)
	Female	146 (97.3)
Age	23–29 years	43 (28.7)
	30–39 years	84 (56.0)
	40–52 years	23 (15.3)
Marital status	Married	111 (74.0)
	Single	39 (26.0)
Number of children	No children	35 (23.3)
	1 to 2 children	84 (56.0)
	3 or more children	31 (20.7)
Educational level	Training college and below	128 (85.3)
	University and above	22 (14.7)
Monthly salary	Less than 2000 GHS	113 (75.3)
	2000–3000 GHS	37 (24.7)
Years as provider	0–5 years	69 (46.0)
	6–10 years	43 (28.7)
	More than 10 years	38 (25.3)
Perceived social status of family growing up	Bottom half	104 (69.3)
	Upper half	46 (30.7)
Perceived social status of self now	Bottom half	59 (39.3)
	Upper half	91 (60.7)
Social mobility	Upward mobility	102 (68.0)
	No change	35 (23.3)
	Downward mobility	13 (8.7)
Religion	Catholic	121 (80.7)
	Methodist/Presby/Anglican	29 (19.3)
Training on interpersonal interactions	No	60 (40.0)
	Yes	90 (60.0)

All providers completed the questions from the two vignettes, but two respondents were excluded because of incomplete data (*N* = 148). The questions for which there were significant differences by SES were introductions, understanding, exaggerating pain, and litigation (Table 2). Close to half (47%) of providers agreed (agree or strongly agree) that the low SES woman was not likely to expect providers to introduce themselves compared to 21% for the high SES woman; and 39% agreed that the low SES woman was not likely to understand explanations compared to 21% for the high SES woman. On the other hand, providers were more likely to agree that the high SES woman was likely exaggerating her pain (23% for low SES and 42% for high SES) and was more likely to sue them if something goes wrong compared to the low SES woman (57% for low SES and 97% for high SES).

For the other items, there were no statistically significant differences by SES, although the direction of association and magnitude of some of the differences are worth noting. For example, in both vignettes, close to half of providers agreed that since the woman came to the facility, it means she has consented to all examinations and treatment (47% for low SES and 51% for high SES). Also, close to half agreed that the provider needs to be stern for the woman to understand the seriousness of the situation (45% for low SES and 46% for high SES), and about one-third agreed that the woman was likely to be uncooperative when it was time to push and would need to be physically restrained (25% for low SES and 34% for high SES). Very few providers agreed that the woman would not need a companion (7% for low SES and 5% for high SES).

More providers stated they would very much want to be a provider for the woman with the lower SES (78%) than the one with the higher SES (66%), but there were no differences in the extent to which they felt connected with the two patients (average feelings of connectedness of about 8 out of 10 for both: Table 2).

Exploratory factor analysis of the eight PCMC perceptions yielded one factor with eigenvalue > 1 for both vignettes (Table S1). The question on litigation had low loadings on the first factor for both vignettes and was dropped. For the response to the low SES vignette, all other items had factor loadings of > 0.3 on the first factor. Three items (introductions, consent, and companion) had loadings between 0.19 and 0.27 on the high SES vignette but were retained based on their conceptual relevance. Cronbach’s alpha for the seven items was 0.65 for the low SES Vignette and 0.61 for the high SES vignette. The hypothetical range of scores on the composite measure from the seven items is from 7 to 28, with higher scores indicating stronger explicit bias. Hypothetically a score of 7 represents no explicit bias and 28 represents the strongest explicit bias. The average explicit bias score was 18.1 (SD = 3.60; range 9–28) for the low SES woman vignette and 16.9 (SD = 3.15; range 8–27) for the high SES woman vignette. Scores did not differ significantly by order of vignette presentation. Mixed-model ANOVA showed a significant difference between the two composite scores (*p* < 0.001), suggesting a significant difference in associating negative perceptions towards the lower SES woman than the higher SES woman. This implies that on average the providers in the sample have stronger negative explicit bias towards the low SES patient than the high SES patient.

#### Implicit SES bias

All providers (*N* = 150) took the IAT. IAT scores ranged from -0.47 to 1.43, with a mean of 0.71 (SD = 0.43; 95%CI = 0.64 to 0.78). Most providers (90.7%) had an IAT score greater than zero. Thus, on average providers in this sample had stronger implicit bias in favor of high SES patients—i.e., bias towards of associating positive characteristics with high SES women and negative characteristics with low SES women.

#### Factors associated with explicit and implicit bias

There was a strong correlation between the explicit bias scores from the two vignettes (*r* = 0.60, *p* < 0.001), but as expected, little correlation between the explicit bias scores and the IAT score (*p* > 0.6) (Table 4). The means bias scores by provider and facility characteristics are presented in Table 4. In the bivariate analysis, only educational status and parity were significantly associated explicit bias. On average providers with lower education had higher scores on the low SES vignette than those with higher education—indicating stronger negative explicit bias towards the low SES woman among providers with lower education than among those with higher education. Providers with higher parity had, on average, stronger negative explicit bias towards the high SES woman than providers with lower parity. For the IAT scores, only facility type was significant in bivariate analysis. On average providers working in government hospitals had lower IAT scores—indicating weaker implicit bias in favor of high SES patients among these providers than those working in government health centers and private facilities (Table 4).

**Table 4:** Bivariate analysis—correlation between provider bias measures and cross tabulations of mean bias scores by provider and facility characteristics

Characteristic	Category	**Score on low SES woman vignette, (*N* = 148)**				**Score on high SES woman vignette, (*N* = 148)**				**IAT score (*N* = 150)**			
		***Correlations***											
		***N***	***r***		***P-value***	***N***	***r***		***P-value***	***N***	***r***		***P-value***
Score on low SES woman vignette						146	0.60		< 0.001	148	0.01		0.946
Score on high SES woman vignette		146	0.60		< 0.001					148	-0.03		0.719
IAT score		150	0.01		0.946	148	-0.03		0.719				
		***Crosstabulations***											
		***N***	***Mean***	***SD***	***P-value***	***N***	***Mean***	***SD***	***P-value***	***N***	***Mean***	***SD***	***P-value***
Total		148	18.07	3.60		148	16.88	3.15		150	0.71	0.43	
Facility type	Govt hospital	91	17.8	3.37	0.511	91	16.9	2.78	0.287	92	0.64	0.43	0.041
	Govt health center	32	18.5	3.92		32	17.4	3.95		32	0.83	0.37	
	Mission/private	25	18.6	4.03		25	16.1	3.26		26	0.80	0.44	
Position	Nurse	129	17.9	3.69	0.146	131	16.8	3.24	0.189	131	0.70	0.43	0.265
	Midwife	11	18.1	2.55		10	16.9	1.97		11	0.90	0.35	
	Ward aid/assistant/support	8	20.5	2.62		7	19.0	2.24		8	0.62	0.44	
Gender	Male	4	18	1.15	0.967	4	16.8	2.50	0.935	4	1.05	0.55	0.108
	Female	144	18.1	3.65		144	16.9	3.18		146	0.70	0.42	
Age	23–29 years	43	18.3	3.24	0.223	43	16.7	2.71	0.066	43	0.67	0.45	0.612
	30–39 years	83	17.7	3.73		83	16.6	3.45		84	0.71	0.42	
	40–52 years	22	19.1	3.68		22	18.3	2.40		23	0.78	0.40	
Marital status	Married	109	18.1	3.73	0.801	110	17.0	3.35	0.361	111	0.68	0.44	0.110
	Single	39	17.9	3.26		38	16.5	2.50		39	0.80	0.37	
Number of children	No children	35	17.9	3.47	0.241	34	16.0	2.53	0.015	35	0.79	0.43	0.318
	1 to 2 children	83	17.8	3.67		84	16.8	3.24		84	0.66	0.43	
	3 or more children	30	19.1	3.51		30	18.2	3.18		31	0.74	0.41	
Educational level	Training College and below	126	18.4	3.37	0.004	126	17.1	3.07	0.075	128	0.70	0.44	0.705
	University and above	22	16.0	4.27		22	15.8	3.46		22	0.74	0.39	
Monthly salary	Less than 2000 GHS	111	18.2	3.57	0.573	111	16.8	3.17	0.742	113	0.70	0.44	0.053
	2000–3000 GHS	37	17.8	3.74		37	17.0	3.14		37	0.83	0.38	
Years as provider	0–5 years	68	18.4	3.22	0.667	68	16.8	3.03	0.443	69	0.69	0.43	0.232
	6–10 years	42	17.7	3.28		43	17.3	2.79		43	0.65	0.43	
	More than 10 years	38	17.9	4.54		37	16.5	3.74		38	0.81	0.41	
Perceived social status of family growing up	Bottom half	102	17.9	3.57	0.388	102	16.9	3.29	0.805	104	0.72	0.40	0.672
	Upper half	46	18.5	3.69		46	16.8	2.85		46	0.69	0.48	
Perceived social status of self now	Bottom half	57	17.8	3.77	0.448	58	16.6	3.23	0.340	59	0.67	0.44	0.387
	Upper half	91	18.3	3.50		90	17.1	3.10		91	0.73	0.42	
Social mobility	Upward mobility	101	17.9	3.51	0.154	100	16.9	3.17	0.808	102	0.73	0.43	0.126
	No change	34	19.1	3.57		35	17.1	3.21		35	0.72	0.36	
	Downward mobility	13	17.2	4.16		13	16.4	3.04		13	0.48	0.57	
Religion	Catholic	120	18.0	3.67	0.490	120	16.9	3.67	0.873	121	0.71	0.42	0.899
	Methodist/Presby/Anglican	28	18.5	3.32		28	17.0	2.50		29	0.70	0.47	
Training on interpersonal interactions	No	60	18.5	3.53	0.219	59	17.2	2.71	0.335	60	0.71	0.44	0.924
	Yes	88	17.8	3.64		89	16.7	3.42		90	0.71	0.42	

Higher mean scores on the low SES vignette indicate stronger negative explicit bias towards the low SES woman, while higher mean scores on the high SES vignette indicate stronger negative explicit bias towards the high SES woman. Higher IAT scores indicate stronger bias in favor of high SES patients

In multivariate analysis (Table 5), education was again significantly associated with low SES bias scores, with those with higher education having a lower score on the low SES vignette than those with lower education—indicating providers with higher education had weaker negative explicit bias towards the low SES woman. Also, providers who reported no change in social mobility had stronger negative explicit bias towards the low SES woman than those who had experienced upward mobility. On the high SES vignette, providers in private facilities had lower bias scores than those in government hospitals, indicating providers in private hospitals had weaker negative explicit bias towards the high SES woman than those in government hospitals. Providers with more than 10 years of experience also had weaker explicit bias toward the high SES woman than those with fewer years of experience. Older age and higher parity were associated with stronger negative explicit bias towards the high SES woman. For implicit bias, providers in private facilities and government health centers had higher IAT scores than those in government hospitals, indicating providers in private facilities and lower level government facilities have stronger implicit bias in favor of high SES women—i.e., associating positive characteristics with high SES women and negative characteristics with low SES women. Also, providers with higher income had stronger implicit bias in favor of high SES women than those with lower income. Compared those who had achieved upward mobility, those who reported downward change in social mobility had stronger implicit bias in favor of low SES women.

**Table 5:** Multivariate analysis of factors associated with provider explicit and implicit bias

Characteristic	Category	Score on low SES woman vignette, (*N* = 148)		Score on high SES woman vignette, (*N* = 148)		IAT score, (*N* = 150)	
		Coeff (95% CI)	*p*-value	Coeff (95% CI)	*p*-value	Coeff (95% CI)	*p*-value
Facility type	Govt hospital			Reference	-	Reference	-
	Govt health center/Dispensary			0.90 (-0.41,2.21)	0.180	0.18 (0.04,0.32)	0.013
	Mission/private			-0.89 (-1.64, -0.15)	0.019	0.18 (0.07,0.29)	0.002
Age	23–29 years			Reference	-		
	30–39 years			0.10 (-1.40,1.59)	0.900		
	40–52 years			2.53 (0.51,4.54)	0.014		
Marital status	Married					Reference	-
	Single					0.15 (-0.02,0.32)	0.091
Number of children	No children			Reference	-		
	1 to 2 children			0.92 (-0.49,2.32)	0.200		
	3 or more children			2.40 (0.55,4.24)	0.011		
Educational level	Training college and below	Reference	-				
	University and above	-2.17 (-3.74, -0.60)	0.007				
Monthly salary	Less than 2000 GHS					Reference	-
	2000–3000 GHS					0.12 (0.01,0.23)	0.041
Years as provider	0–5 years			Reference	-		
	6–10 years			-0.35 (-2.07,1.38)	0.694		
	More than 10 years			-2.87 (-4.87, -0.88)	0.005		
Social mobility	Upward mobility	Reference	-			Reference	-
	No change	1.11 (0.04,2.19)	0.043			-0.04 (-0.17, 0.08)	0.493
	Downward mobility	-0.51 (-1.99,0.97)	0.500			-0.24 (-0.43, -0.05)	0.013
Training on interpersonal interactions	No	Reference	-				
	Yes	-0.57 (-1.41,0.28)	0.189				

Higher mean scores on the low SES vignette indicate stronger negative explicit bias towards the low SES woman, while higher mean scores on the high SES vignette indicate stronger negative explicit bias towards the high SES woman. Higher IAT scores indicate stronger implicit bias in favor of high SES patients

## Discussion

This study provides further evidence of explicit and implicit biases among maternity providers that could lead to disparities in PCMC based on SES. When presented with vignettes representing a woman of low SES and one of high SES, overall, providers had more negative perceptions about the low SES woman manifested in their higher agreement to statements such as the low SES woman is not likely to expect providers to introduce themselves and not likely to understand explanations when compared to responses about the high SES woman. On the other hand, they were more likely to agree that the high SES woman was likely exaggerating her pain and was more likely to sue them if something went wrong, compared to responses for the low SES woman. Further, providers overall, showed implicit bias between women’s SES and their perceptions of those women as good or difficult patients. Specifically, providers were more likely to associate higher SES characteristics with good attributes and lower SES with negative attributes than the reverse. Such perceptions likely contribute to the poorer PCMC experiences among women of lower SES.

To our knowledge, this is the second study to examine provider implicit biases in PCMC in SSA and the first in Ghana. The current study and the previous one in Kenya provide consistent evidence on the role of implicit bias in a context where the predominant bias is not racial bias. As has been previously noted, bias (implicit and explicit) is prevalent in every society, although the content of biases may differ across different contexts [35]. For instance, in contexts like the US, racial bias is a key contributor to health disparities. SES bias likely plays a greater role in other settings where there is less racial diversity (as socially constructed), and social class is an important determinant of how people are treated in societies. The significant bias in favor of associating positive characteristics with high SES women and negative characteristics with low SES women likely influences how providers interact with each group. Prior research in Kenya, showed that providers sometimes unconsciously treated higher SES women better based on their attractions to their physical appearance, although they did not necessarily prefer higher SES women as patients [39]. This is consistent with the findings here, where there were no differences in the extent to which they reported feeling connected with the two patients in the vignettes, despite more negative implicit bias towards the lower SES woman.

This study provides stronger evidence on the role of explicit SES bias in PCMC. Unlike the Kenya study, where we did not find statistically significant overall differences in the composite explicit bias scores for the two vignettes, we did observe statistically significant differences supporting more negative biases towards low SES women in this study. This is likely because of the larger sample size and bigger differences in the magnitude of the associations for the individual items. In both studies, providers were more likely to agree that the low SES woman is not likely to expect providers to introduce themselves and is not likely to understand explanations, compared to the high SES woman. These likely explain SES differences in PCMC. If providers perceive less expectation of self-introduction and lower capacity for understanding explanations in low-SES patients, less information-giving and relationship-building is likely to follow. This has been previously documented in studies with both patients and providers in Kenya [12, 39, 57]. In other analysis, only 21% of providers in this sample reported always introducing themselves and 49% reported always explaining the purpose of examinations and procedures to their patients, which is informative given they serve predominantly low SES women [58]. Similarly, in both studies, providers were more likely to agree that the high SES woman is likely exaggerating her pain and is more likely to sue them if something goes wrong compared to the low SES woman. Such perceptions may lead to pain medication being withheld from high SES women who genuinely need it. Higher SES women may however still obtain adequate pain medication and experience more positive person-centered care because they are able to demand and advocate for their needs and are perceived to have the means to pursue legal redress [57, 66]. On the other hand, poorer women may be treated negligently because of the perception that they will be unable to seek legal redress. Women’s ability to advocate for themselves or hold providers accountable is a key factor in how they are treated [55, 57, 66].

In both the current and the prior Kenya study, close to half of providers agreed that since the woman came to the facility, it meant she had consented to all examinations and treatment and that the provider needed to be stern for women to understand the seriousness of the situation. Further, about one-third of providers in both the Ghana and Kenya samples agreed that women were likely to be uncooperative when it was time to push and would need to be physically restrained. Such perceptions likely contribute to findings from previous studies in this setting where women reported experiences of providers not asking for consent before doing examinations and procedures on them and providers being rude and physically abusive [8, 12, 67]. Providers have also reported using physical and verbal abuse as a means of gaining compliance during difficult situations [55, 57, 68]. These findings, though not necessarily indicative of bias, are likely a reflection of the common training, experiences, and health system culture, and need to be addressed in interventions to improve PCMC. Intervention strategies should include training providers on the importance of consenting and patient autonomy, how to communicate complications to patients in a respectful and supportive manner and how to handle difficult situations where patients may not be as compliant. Training should be accompanied by strategies to motivate and support providers, reinforce positive behaviors, as well as well as strategies to hold them accountable for negative behaviors.

Interestingly, more providers stated they would want to be a provider for the woman with lower SES than the one with the higher SES. This is consistent with qualitative data, which illuminates the contradictory ways various factors influence provider behavior. Providers preferred to care for lower SES patients because they often “did what they were told,” but ended up providing poorer care to them because they were perceived to be less likely to understand what they were told, had lower expectations, were less likely to advocate for themselves, and were less likely to hold them accountable [39, 55, 57]. Interventions that educate low SES patients on their rights and empower them to communicate their expectations and hold providers accountable may enable them to advocate for better care. However, such interventions place the onus of receiving good care on the patient and not the provider. Provider and system level interventions are thus required, as discussed subsequently.

We found associations between some provider socio-demographic factors and explicit bias scores in this and the prior Kenya study. This is not surprising given that reports on PCMC perceptions are influenced by knowledge, which in turn are influenced by socio-demographic factors like education [36, 37]. But unlike in the Kenya study, where no provider characteristics were associated with implicit bias, we found some associations between implicit bias and facility type as well as some provider socio-demographic factors. The role of facility type is especially important given prior research suggests women who give birth in private facilities receive better PCMC [8, 21, 25]. Given people who seek care in these private facilities are more likely to be of higher SES, higher care in these facilities may be due to a combination of factors including bias in favor of higher SES patients. This means that low SES patients seeking care in these private facilities may still receive poorer care if other factors such as higher accountability at the institutional level are not enforced. Such accountability measures include creating mechanisms for all patients to provide feedback on their care experience and providing opportunities for redress.

Studies on SES bias in high income countries such as the United States also support the findings presented here. Health visits with lower-SES patients are found to have less time spent on patient questions and assessment of patient’s health knowledge, as well as less socioemotional support and partnership-building conversations [69]. Furthermore, implicit bias towards low-SES patients (and resultant implicit preference towards high-SES patients) has been documented in studies of healthcare professionals in these settings [37, 70, 71]. Research indicates that implicit bias towards low-SES patients may translate into poorer person-centered care, with low-SES patients experiencing less involvement in treatment decisions and lower control over communication [72]. Across studies, lower-SES patients report that their providers communicate poorly, thereby failing to exhibit a core tenet of person-centered care [73, 74].

Our findings imply a need for multilevel interventions to address both implicit and explicit provider biases to reduce the disparities in PCMC. Prior research has used strategies such providing lists of questions for patients to ask doctors during health visits [75] and using coaching to teach communication skills to patients [76], as a way of empowering low SES patients to communicate their expectations and advocate for themselves. As noted however, such interventions place the onus of receiving good care on the patient and not the provider, which should not be the case. Further, requiring time from low-SES patients over and above that spent seeking care to increase their chances of receiving good care is certain to present additional challenges to patients who may already be dealing with several challenges.

An alternative and perhaps more effective avenue for designing interventions to improve outcomes for low-SES patients is to target healthcare providers. Training providers to recognize their biases, to be *concerned* about the effects of bias, to be motivated to identify and learn to replace biased response with responses more consistent with their goals, have been shown to be effective in reducing racial bias [77, 78]. Further, emerging research suggests that it is possible to reduce the effects of people’s bias through activities that elevate the alternative selves and goals that people endorse, without actually removing their deep-seated biases—referred to as sidelining bias [79]. For example, when probation officers adopt a mindset focused on reaching their professional goals to help people get back on their feet—especially people who may not receive that support elsewhere due to previous incarceration—biases against those stigmatized people are rendered dysfunctional to those officers’ reaching their goals; and in turn, mitigate disparities in life outcomes (e.g., recidivism to jail) for previously incarcerated people the officers supervise [80]. Likewise, healthcare providers can be strategically reminded of their professional goals to help people—especially those most in need and unable to otherwise get support—in a way that would render bias and its consequences dysfunctional. Interventions can thus shape healthcare provider mindsets towards empathy. Such interventions have also sidelined consequences of teachers’ biases against students from stigmatized groups and mitigated disparate outcomes in discipline that remove students from the learning environment [80–83]. Evaluations in health care settings are however needed. Beyond these, there is a need to create structures to minimize the effects of people’s individual biases [34]. These can include institutional policies around introductions, communicating procedures, consenting, pain management, among others, and institutional structures for accountability.

### Limitations and strengths

First, bias is not a socially desirable attitude, provider’s responses to the questions on explicit bias is thus likely influenced by their perceptions of what they think is the right answer leading to social desirability bias. However, the variation in responses including evidence of bias suggest that providers are willing to explicitly note their bias based on SES characteristics. The relatively low Cronbach alpha for the explicit bias measures is also a limitation. Second, the predictive validity of the IAT in terms of predicting behavior remains disputed, with lack of clarity on whether implicit bias would translate to behavioral differences towards patients among health care professionals [84–86]. Studies examining such relationships are needed. Finally, our sample is drawn from providers (mostly nurses and midwives) working in high-volume maternity units in one region in Ghana and thus may not be generalizable to all providers. Nonetheless, this study makes an important contribution to the literature to achieve equity in PCMC. It is one of the few studies on sources of PCMC disparities in a low-resource setting, and only the second to examine implicit bias in SSA.

## Conclusions

The findings from this study strengthens the evidence on the presence of both implicit and explicit SES bias among maternity providers in SSA. This study is important given the dearth of research on how to improve PCMC for low SES patients. The findings provide insights on alternative interventions to achieve equity in PCMC. Such interventions can be approached at different levels including increasing low SES women’s ability to advocate for themselves and interventions that target providers attitudes, mindset, and behavior. Lasting change will however likely come from health system interventions that both motivate and hold providers accountable for equity in PCMC, as well as strengthen the overall health system. Research to develop and test such interventions are urgently needed to reduce disparities in PCMC and to improve PCMC for all women as part of efforts to achieve the fundamental human right of dignity and respect and to achieve the global goals of reducing inequities in maternal mortality and morbidity.

## Supplementary Information

**Additional file 1.**

**Additional file 2.**

## Data Availability

The data supporting the conclusions of this article are included within the article and in additional file 1.
