# Supplementary material for: Provider implicit and explicit bias in person-centered maternity care: a cross-sectional study with maternity providers in Northern Ghana
Source: BMC Health Serv Res. 2023 Mar 14;23:254. doi: 10.1186/s12913-023-09261-6 (PMC10015736; doi:10.1186/s12913-023-09261-6)
Supplement: Supplementary file 1 — Additional file 1. [file 12913_2023_9261_MOESM1_ESM.docx]

| **Table S1: Results from exploratory factor analysis of the 8 explicit bias questions** | | | | | |
| --- | --- | --- | --- | --- | --- |
| Responses to Low SES vignette | | | | | |
| Variable | Count | Mean (SD) | Min | Max |  |
| Not likely to expect introduction | 150 | 2.40 (0.84) | 1 | 4 |  |
| Not likely to understand any explanations | 150 | 2.34 (0.99) | 1 | 4 |  |
| Has consented for all | 149 | 2.50 (0.95) | 1 | 4 |  |
| Likely exaggerating her pain | 150 | 2.08 (0.82) | 1 | 4 |  |
| Not need a companion to stay with her | 150 | 1.80 (0.65) | 1 | 4 |  |
| Stern for her understand the seriousness | 150 | 2.38 (0.89) | 1 | 4 |  |
| Uncooperative when it is time to push | 150 | 2.15 (0.85) | 1 | 4 |  |
| Likely to sue you if something goes wrong | 149 | 2.34 (0.88) | 1 | 4 |  |
| Average interitem correlation | | | 0.1789 |  |  |
| Number of items in the scale | | | 8 |  |  |
| Scale reliability coefficient | | | 0.6355 |  |  |
|  | | | | | |
| Factor | Eigenvalue | Difference | Proportion | Cumulative |  |
| Factor 1 | 1.59543 | 1.15706 | 0.9934 | 0.9934 |  |
| Factor 2 | 0.43836 | 0.12211 | 0.2730 | 1.2664 |  |
| Factor 3 | 0.31625 | 0.27751 | 0.1969 | 1.4633 |  |
| Factor 4 | 0.03874 | 0.19901 | 0.0241 | 1.4874 |  |
| Factor 5 | -0.16027 | 0.03441 | -0.0998 | 1.3876 |  |
| Factor 6 | -0.19468 | 0.01573 | -0.1212 | 1.2664 |  |
| Factor 7 | -0.21041 | 0.00703 | -0.1310 | 1.1354 |  |
| Factor 8 | -0.21744 | - | -0.1354 | 1.0000 |  |
| Factor loadings retaining 3 factors | | | | |  |
| Variable | Factor 1 | Factor 2 | Factor 3 | uniqueness |  |
| Not likely to expect introduction | 0.3441 | 0.3840 | 0.0354 | 0.7329 |  |
| Not likely to understand any explanations | 0.6361 | 0.0868 | -0.1934 | 0.5504 |  |
| Has consented for all | 0.2963 | -0.2256 | -0.1860 | 0.8267 |  |
| Likely exaggerating her pain | 0.5807 | 0.2568 | -0.1072 | 0.5854 |  |
| Not need a companion to stay with her | 0.3347 | -0.0638 | 0.3403 | 0.7681 |  |
| Stern for her understand the seriousness | 0.5624 | -0.2364 | 0.1031 | 0.6172 |  |
| Uncooperative when it is time to push | 0.4386 | -0.3056 | 0.0077 | 0.7142 |  |
| Likely to sue you if something goes wrong | 0.1635 | 0.1147 | 0.3240 | 0.8551 |  |
|  |  |  |  |  |  |
| Responses to High SES vignette | | | | |  |
| Variable | Count | Mean (SD) | Min | Max |  |
| Not likely to expect introduction | 148 | 2.11 (0.76) | 1 | 4 |  |
| Not likely to understand any explanations | 149 | 1.92 (0.79) | 1 | 4 |  |
| Has consented for all | 149 | 2.60 (0.94) | 1 | 4 |  |
| Likely exaggerating her pain | 149 | 2.36 (0.86) | 1 | 4 |  |
| Not need a companion to stay with her | 149 | 1.74 (0.54) | 1 | 3 |  |
| Stern for her understand the seriousness | 149 | 2.45 (0.90) | 1 | 4 |  |
| Uncooperative when it is time to push | 149 | 2.29 (0.88) | 1 | 4 |  |
| Likely to sue you if something goes wrong | 149 | 1.44 (0.59) | 1 | 4 |  |
| Average interitem correlation | | | 0.1462 |  |  |
| Number of items in the scale | | | 8 |  |  |
| Scale reliability coefficient | | | 0.5781 |  |  |
|  |  |  |  |  |  |
| Factor | Eigenvalue | Difference | Proportion | Cumulative |  |
| Factor 1 | 1.52352 | 0.92465 | 0.9026 | 0.9026 |  |
| Factor 2 | 0.59886 | 0.37951 | 0.3548 | 1.2574 |  |
| Factor 3 | 0.21936 | 0.10607 | 0.1300 | 1.3874 |  |
| Factor 4 | 0.11328 | 0.18851 | 0.0671 | 1.4545 |  |
| Factor 5 | -0.07523 | 0.07507 | -0.0446 | 1.4100 |  |
| Factor 6 | -0.15030 | 0.10122 | -0.0890 | 1.3209 |  |
| Factor 7 | -0.25152 | 0.03860 | -0.1490 | 1.1719 |  |
| Factor 8 | -0.29012 | - | -0.1719 | 1.0000 |  |
| Factor loadings retaining 3 factors |  |  |  |  |  |
| Variable | Factor 1 | Factor 2 | Factor 3 | uniqueness |  |
| Not likely to expect introduction | 0.2734 | 0.4035 | -0.1922 | 0.7255 |  |
| Not likely to understand any explanations | 0.4876 | 0.1421 | 0.0267 | 0.7414 |  |
| Has consented for all | 0.1952 | -0.2845 | 0.0789 | 0.8748 |  |
| Likely exaggerating her pain | 0.6223 | -0.0460 | -0.1788 | 0.5787 |  |
| Not need a companion to stay with her | 0.2275 | 0.4838 | 0.1099 | 0.7021 |  |
| Stern for her understand the seriousness | 0.5907 | -0.1758 | 0.1447 | 0.5992 |  |
| Uncooperative when it is time to push | 0.6117 | -0.1276 | 0.0948 | 0.6006 |  |
| Likely to sue you if something goes wrong | -0.1038 | 0.2271 | 0.3186 | 0.8361 |  |
